# Optical Coherence Tomography Angiography Compared With Optical Coherence Tomography for Detection of Early Glaucoma With High Myopia

**DOI:** 10.3389/fmed.2021.793786

**Published:** 2022-01-11

**Authors:** Pei-Yao Chang, Jiun-Yi Wang, Jia-Kang Wang, Tzu-Lun Huang, Yung-Ray Hsu

**Affiliations:** ^1^Department of Ophthalmology, Far Eastern Memorial Hospital, New Taipei City, Taiwan; ^2^Department of Medicine, National Taiwan University Hospital, Taipei, Taiwan; ^3^Department of Healthcare Administration, Asia University, Taichung, Taiwan; ^4^Department of Electrical Engineering, Yuan Ze University, Taoyuan City, Taiwan; ^5^Department of Medical Research, China Medical University Hospital, China Medical University, Taichung, Taiwan; ^6^Department of Healthcare Administration and Department of Nursing, Oriental Institute of Technology, New Taipei City, Taiwan

**Keywords:** optic nerve head vessel density, retinal vessel density, retinal nerve fiber layer thickness, open angle glaucoma, high myopia

## Abstract

**Purpose:** To investigate the diagnostic abilities of the perfusion density (PD) and structural thickness parameters in the peripapillary and macular regions measured by optical coherence tomography angiography (OCTA) and optical coherence tomography (OCT) and to test if their diagnostic abilities of early glaucoma are different between highly myopic (HM) and non-highly myopic (NHM) patients.

**Methods:** A total of 75 glaucoma patients and 65 controls were included in the analyses. The glaucoma detection abilities of macular PD and peripapillary PD, along with macular ganglion cell-inner plexiform layer (mGCIPL) thickness and peripapillary retinal nerve fiber layer (pRNFL) thicknesses were compared between the HM and NHM group. Diagnostic ability was assessed by area under the receiver operating characteristics (AUC) curves, adjusted by age, axial length, and signal strength.

**Results:** The diagnostic ability of macular PD and mGCIPL thickness had no significant difference in both HM and NHM groups. However, the diagnostic ability of peripapillary PD except in the temporal section was significantly lower in the HM group than in the NHM group (all *p* < 0.05). The diagnostic ability of the superior, nasal, and average pRNFL thickness was also significantly lower in the HM group than in the NHM group (all *p* < 0.05).

**Conclusion:** This study demonstrated that although peripapillary PD and macular PD were both significantly reduced in patients with highly myopia, the diagnostic ability of peripapillary PD in HM patients was significantly lower than that in NHM patients, while macular PD was not. Macular OCTA along with OCT imaging should be included in the imaging algorithm in early glaucoma diagnosis in highly myopic patients.

## Introduction

Myopia is highly prevalent in Asia and high myopia has been considered an independent risk factor for glaucoma (1). Optic disc of highly myopic patients manifests structural change that mimics glaucoma. Early glaucoma diagnosis is crucial but challenging in myopic patients even for glaucoma specialists because the optic disc of myopic, especially highly myopic patients is usually accompanied by tilting/torsional, shallow cup, and peripapillary atrophy (2). Several studies have reported that myopic eyes have a thinner retinal nerve fiber layer (RNFL) and the pattern of peripapillary retinal nerve fiber layer (pRNFL) defect in high myopic glaucoma is different (3–5). The papillomacular bundle in highly myopic (HM) patients is more susceptible (4, 5) and the macular ganglion cell complex or ganglion cell-inner plexiform layer (GCIPL) thickness was reported to have comparable or even better glaucoma detection ability than pRNFL thickness in HM (6, 7). However, even with the pRNFL and macular GCIPL thickness measurement by spectral-domain optical coherence tomography (SD-OCT), it is still more difficult to detect an early glaucomatous change in highly myopic eyes than in emmetropic eyes (8, 9).

Vessel density parameters measured by OCT angiography (OCTA) in the peripapillary and macular regions in the glaucomatous eyes were significantly lower than those in the healthy eyes (10–13). Peripapillary/macular vessel density changes have been reported to be useful for differentiating between glaucoma and healthy eyes, especially in HM patients because vessel density measurement is less affected by the low reflectance of the RNFL or optic disc deformation (14, 15). That is, if the vessel density changes are more prominent than other structural changes at the early stage of glaucoma in HM, it would be helpful for this challenging early glaucoma diagnosis. Thus, we focused on the vessel density parameters in HM patients with early glaucoma to investigate the diagnostic abilities of the vessel density parameters in the peripapillary and macular regions and to test if their diagnostic abilities are different between HM and non-highly myopic (NHM) patients. We further compared their diagnostic abilities of structural OCT parameters, such as pRNFL and macular GCIPL thickness, with vessel density parameters to investigate their performance in early glaucoma diagnosis.

## Methods

### Study Design and Procedure

This retrospective cross-sectional study collected medical records of patients who visited the glaucoma clinic in a medical center in northern Taiwan between January and June 2018. The inclusion criteria for all groups were age more than 20 years, best-corrected visual acuity ≥20/30, and open-angle confirmed by gonioscopy. Glaucomatous eyes were defined as glaucomatous damage to the optic disc as accompanied by two corresponding and reliable abnormal visual field (VF) examinations, regardless of the intraocular pressure (IOP). Subjects with evidence of retinal pathology, diabetes, hypertension, or non-glaucomatous optic nerve diseases were excluded, as well as eyes that had undergone previous laser therapy or ocular surgery, or the presence of any media opacities that prevented high-quality OCT scans. If both eyes of a patient were eligible, one eye was selected at random.

The study was performed in accordance with the tenets of the Declaration of Helsinki and was approved by the Institutional Review Board at Far Eastern Memorial Hospital (No. 107135-E). Informed consent was waived as personal identities in the retrospective data were recoded and delinked.

### Measures

All of the subjects underwent a complete ophthalmic examination, which included measurement of visual acuity, IOP measurement through noncontact tonometry (CT-80, Topcon, Japan), refractive error measurement through autorefraction (Auto Refractometer AR-610; Nidek Co, Ltd., Tokyo, Japan), slit-lamp examination; gonioscopy; dilated fundus examination with simultaneous stereophotography of the optic disc, and red-free RNFL photography. VF testing was performed using a Humphrey Field Analyzer (SITA full threshold programs 30-2; Carl Zeiss Meditec, Inc., Dublin, CA, USA).

The refraction data were converted to spherical equivalents (SE), and the subjects were divided into two groups: an HM group (spherical equivalent < -6.00 diopters [D] and >-13.00 D) and an NHM group (spherical equivalent ≥ −6.00 D and < +0.5 D).

Glaucomatous optic disc changes were defined on stereoscopic color disc photography as a large cupping (>0.7 vertical cup/disc ratio), cup/disc asymmetry between the glaucomatous and normal eyes >0.2, neuroretinal rim thinning, notching, or excavation. Eyes with glaucomatous VF defects were defined as those with a glaucoma hemifield test result outside normal limits or a pattern standard deviation outside 95% of normal limits. Additionally, a cluster of three points with probabilities of 5% on the pattern deviation map in at least one hemifield, such as at least one point with a probability of 1%, or a cluster of two points with a probability of 1% was needed. A VF was defined as reliable when fixation losses were <20%, and each of the false-positive and false-negative rates was <15%. Normal eyes were defined as IOP <21 mm Hg, an absence of glaucomatous optic disc appearance as determined by two masked observers, and no perimetric defects.

Peripapillary (Optic Disc Cube 200 × 200 protocol) and macular (Macular Cube 512 × 128 protocol) scans (collectively referred to as ganglion cell analysis) were acquired using the Cirrus 5000 HD-OCT (Carl Zeiss Meditec, Inc.). Software released by the manufacturer was used to calculate pRNFL and mGCIPL thicknesses, as previously described (16). The pRNFL thickness was measured in a circle of 3.46 mm in diameter and the circumpapillary average, superior, inferior, temporal, and nasal quadrant thicknesses were analyzed. Regarding the macular GCIPL thickness, average and sectoral (superonasal, superior, superotemporal, inferotemporal, inferior, and inferonasal) were analyzed.

**Figure 1:**
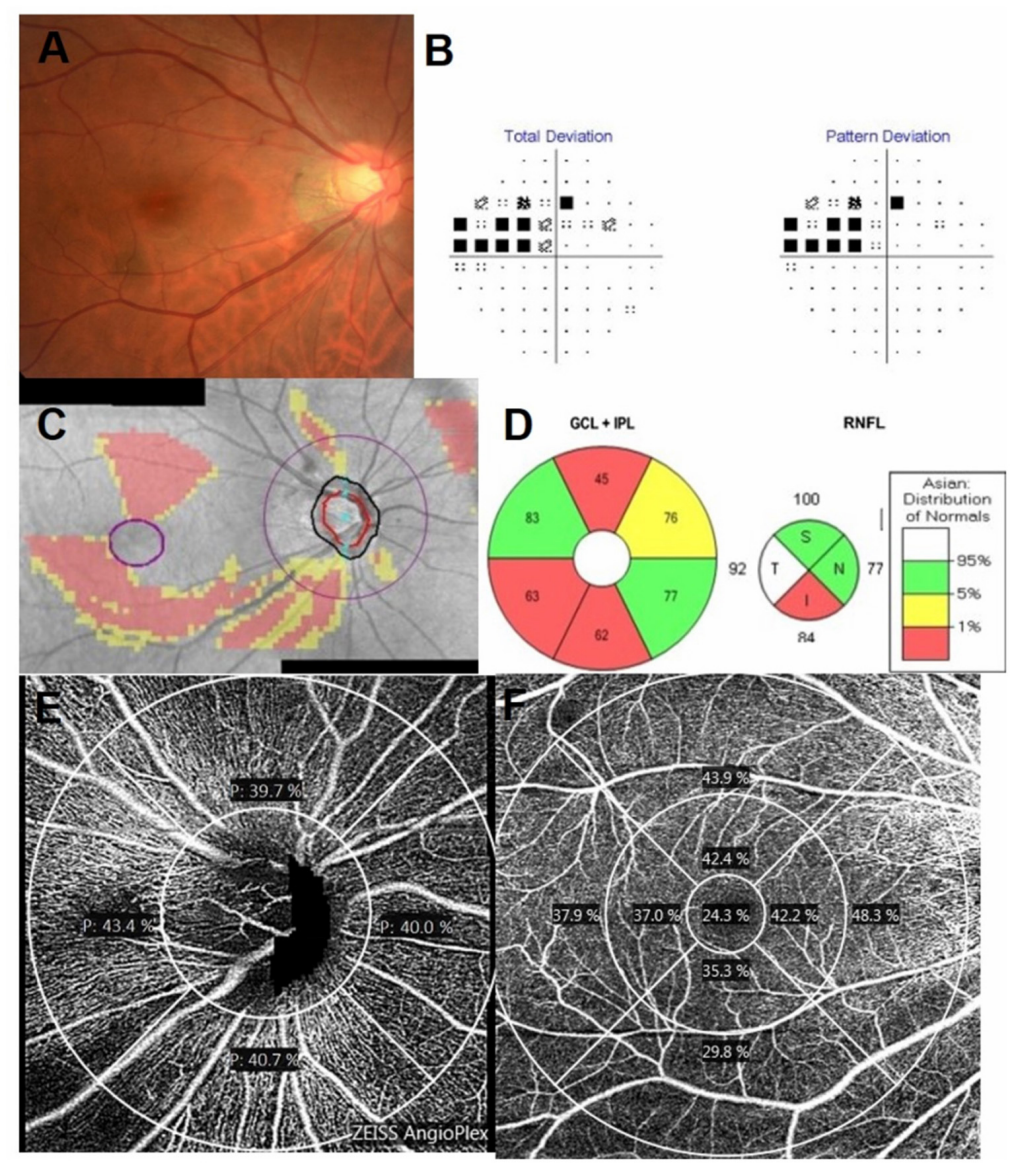
Inferior disc excavation with inferior nerve fiber layer wedge defect of the right eye of a 42-year-old man with POAG **(A)**. Visual field (VF) results revealed defects at the corresponding superonasal site **(B)**. Combined retinal nerve fiber layer (RNFL) and GCIPL deviation maps indicated structural glaucomatous damage **(C)**. OCT scan showed the GCIPL thickness (μm) provided by the ganglion cell analysis report [**(D)**, left] and RNFL thickness (μm) (**(D)**, right) in each sector. OCT angiography image (Cirrus HD-OCT, version 11) of a 4.5 mm × 4.5 mm scan **(E)** exhibited radial peripapillary superficial perfusion (%) and a 6 mm × 6 mm scan **(F)** exhibited the superficial vessels at the macula. The grids represented the sectors across which the perfusion density (%) was calculated. The area between the inner two circles in the 6 mm × 6 mm scan represented the inner sectors, and the area between the outer two circles represented the outer sectors. GCIPL, ganglion cell-inner plexiform layer; OCT, optical coherence tomography; I, inferior; IN, inferonasal; IT, inferotemporal; N, nasal; S, superior; SN, superonasal; ST, superotemporal; T, temporal.

Optical coherence tomography angiography imaging of the radial peripapillary and macular capillaries was performed using the AngioPlex™ (Cirrus; Zeiss, Dublin, USA; software Version 11). Angiographic images were generated through OCT-based microangiography (OMAG) and the procedure for OCTA imaging using the Cirrus HD-OCT has been detailed previously (17). The optic nerve scan was imaged using a 4.5 mm^2^ × 4.5 mm^2^ scan and the macula scan using a 6 mm^2^ × 6 mm^2^ scan pattern. Angiometric software of the Cirrus HD-OCT automatically calculates perfusion density (defined as the total area of perfused vasculature per unit area in the region of interest) from the superficial retinal layer slab and radial outer region peripapillary capillaries. This software calculates the macular perfusion density parameters across four inner and four outer sectors of the Early Treatment Diabetic Retinopathy Study (ETDRS) grid over the macula. Inner and outer ring measurements were analyzed by sectoral locations (inferior, superior, nasal, and temporal). The software also calculates the radial peripapillary perfusion density across the four sectors (inferior, superior, nasal, and temporal) (Figure 1).

Image quality was assessed for all OCTA and OCT scans. Poor quality images, which were defined as those with a signal strength of <7, poor centration, or motion artifacts and segmentation errors, were excluded from the analysis.

### Statistical Analysis

Data were analyzed using IBM SPSS version 22.0 (Armonk, NY: IBM Corp.). Descriptive statistics like the number for categorical data and the mean ± standard deviation for continuous data were used to demonstrate characteristics of the study participants and distributions of the biomarkers. A two-sample *t*-test was conducted to compare the variable distributions between controls and glaucoma cases in the HM group and in the NHM group, as well as between controls or between glaucoma cases in the two groups. Multivariable logistic regression models which included one major biomarker and adjusted for sex, age, and SE and signal strength were first conducted to estimate the likelihood of being glaucoma for the myopia group and the non-myopia group. Then the area under the receiver operating characteristic curves (AUCs) was calculated and compared between the two groups. The comparison between AUCs was based on the method demonstrated in Gonen (18). The significance level was set at 0.05 for all analyses.

## Results

In total, 181 individuals (81 normal and 100 primary open angle glaucoma patients) underwent OCTA measurements. Among them, 10 eyes (5.5%) with motion artifacts in OMAG scans, 20 eyes (11.05%) with a signal strength of <7, 4 eyes (2.2%) with poor structural scans of optic nerve head OCT images owing to decentration and 7 eyes (3.9%) with media opacity, such as vitreous floaters, were excluded. Finally, 140 eyes, comprising 65 healthy subjects and 75 glaucoma patients, were included in the analysis. All participants in the glaucoma group were treated with at least one type of ocular antihypertensive medication at the time of OCTA imaging. The demographic and ophthalmic characteristics of the four groups are summarized in Table 1. The mean age among NHM glaucoma patients was significantly higher than that in NHM control subjects, but the age was not significantly different in the HM group. The mean VF defect had no significant difference between the NHM glaucoma group (mean [SD], 2.98 [2.12]) and the HM glaucoma group (mean [SD], 2.73 [2.22]).

**Table 1:** Baseline characteristics of the control and glaucoma groups.

	**Highly myopic**,			**Non-highly myopic**,				
	***n*** **=** **70**			***n*** **=** **70**				
	**Control,** ***n*** **= 27**	**Glaucoma,** ***n*** **= 43**	***P***	**Control,** ***n*** **= 38**	**Glaucoma,** ***n*** **= 32**	***P***	***P*** **^†^**	***P*** **^‡^**
Age (y)	39.63 ± 7.63	42.63 ± 10.72	0.210	45.18 ± 8.56	54.94 ± 8.65	<0.001	0.009	<0.001
Sex (Male:Female)	11:16	28:15	0.046	21:17	24:8	0.086	0.248	0.359
SE (D)	−8.87 ± 2.00	−9.12 ± 2.30	0.642	−1.87 ± 2.05	−2.70 ± 2.01	0.091	<0.001	<0.001
Visual field index (%)	99.25 ± 0.99	94.30 ± 4.68	<0.001	99.30 ± 0.95	93.25 ± 4.98	<0.001	0.839	0.352
Mean deviation (dB)	−0.50 ± 1.42	−2.73 ± 2.22	<0.001	−0.12 ± 1.07	−2.98 ± 2.12	<0.001	0.262	0.621
Optic Disc area (*mm*^2^)	1.84 ± 0.48	1.92 ± 0.56	0.526	2.19 ± 0.42	2.00 ± 0.56	0.109	0.002	0.539
Rim Area (mm)	1.23 ± 0.30	0.88 ± 0.24	<0.001	1.63 ± 2.44	0.82 ± 0.20	0.065	0.405	0.245
Average C/D ratio (%)	0.54 ± 0.12	0.70 ± 0.13	<0.001	0.62 ± 0.15	0.75 ± 0.07	<0.001	0.032	0.024
IOP at the scanning visit (mm Hg)	17.44 ± 4.02	15.72 ± 3.15	0.049	18.46 ± 4.61	14.69 ± 2.50	<0.001	0.358	0.130

† *Value for comparison between normal and glaucomatous eyes in the non-highly myopic group*.

‡ *Value for comparison between highly and non-highly myopic groups*.

The macular perfusion density, comparing the normal and glaucomatous eyes in both the HM and NHM groups, was significantly different (all *p* < 0.05), except for the inner nasal (*p* = 0.103) in the HM group and inner nasal, inner temporal, and inner superior section in the NHM group. The optic disc perfusion, comparing the normal and glaucomatous eyes in both the HM and the NHM groups, was significantly different (all *p* < 0.05), except for the nasal (*p* = 0.103) in the HM group (Table 2). There was no significant difference in all sections of the macular perfusion density and the optic disc perfusion between the glaucoma patients in the HM group and those in the NHM group. The signal strength of the macular perfusion density, macular GCIPL thickness, pRNFL thickness, except for the optic disc perfusion was significantly higher in normal controls than in the glaucoma patients. The mGCIPL thickness in every section, except the temporal section in the HM group, was significantly thicker in controls than in the glaucomatous patients, whether in the NHM group or in the HM group. The pRNFL thickness in every section was significantly thinner in glaucomatous eyes than in controls, both in highly and non-highly myopic eyes (all *p* < 0.05, Table 2). The AUCs of structural and vascular parameters are shown in Table 3. The diagnostic ability of macular perfusion density and mGCIPL thickness had no significant difference between the HM and the NHM group. However, the diagnostic ability of optic disc flux index in every section and perfusion density except in the temporal section was significantly lower in the HM group than in the NHM group. The diagnostic ability of the superior, nasal, and average pRNFL thickness was also significantly lower in the HM group than in the NHM group. However, when comparing the AUCs of OCT and OCTA measurement, the diagnostic performance had no significant difference between outer region mean perfusion density/flux index and average pRNFL thickness and between outer mean macular perfusion density and average mGCIPL thickness (Table 4).

**Table 2:** Vessel perfusion density parameters measured by optical coherence tomography angiography (OCTA) and structural parameters measured by optical coherence tomography (OCT).

	**Highly myopic**			**Non-highly myopic**				
	***n*** **= 70**			***n*** **= 70**				
	**Control** ***n*** **= 27**	**Glaucoma** ***n*** **= 43**	***P***	**Control** ***n*** **= 38**	**Glaucoma** ***n*** **= 32**	***P***	***P*** **^†^**	***P*** **^‡^**
**Macula perfusion density**								
Signal strength	8.7 ± 1.33	7.7 ± 1.26	0.002	8.84 ± 1.29	8.09 ± 1.2	0.015	0.674	0.175
Outer superior	0.45 ± 0.04	0.39 ± 0.07	<0.001	0.44 ± 0.04	0.38 ± 0.06	<0.001	0.781	0.856
Outer inferior	0.44 ± 0.05	0.33 ± 0.07	<0.001	0.44 ± 0.04	0.34 ± 0.08	<0.001	0.992	0.474
Outer temporal	0.39 ± 0.07	0.31 ± 0.09	0.001	0.39 ± 0.06	0.33 ± 0.07	<0.001	0.698	0.570
Outer nasal	0.49 ± 0.03	0.45 ± 0.07	0.001	0.48 ± 0.03	0.45 ± 0.05	0.006	0.210	0.884
Inner superior	0.43 ± 0.04	0.38 ± 0.1	0.006	0.41 ± 0.06	0.38 ± 0.07	0.138	0.141	0.855
Inner inferior	0.41 ± 0.06	0.35 ± 0.09	0.001	0.41 ± 0.05	0.38 ± 0.07	0.041	0.691	0.215
Inner temporal	0.4 ± 0.04	0.34 ± 0.1	0.002	0.4 ± 0.06	0.37 ± 0.07	0.123	0.894	0.157
Inner nasal	0.41 ± 0.05	0.38 ± 0.09	0.103	0.41 ± 0.05	0.4 ± 0.06	0.256	0.782	0.410
Inner mean	0.41 ± 0.04	0.37 ± 0.08	0.003	0.41 ± 0.05	0.38 ± 0.06	0.077	0.611	0.368
Outer mean	0.44 ± 0.04	0.37 ± 0.06	<0.001	0.44 ± 0.03	0.37 ± 0.05	<0.001	0.615	0.760
Full mean	0.43 ± 0.04	0.36 ± 0.06	<0.001	0.42 ± 0.04	0.37 ± 0.05	<0.001	0.608	0.661
**Optic disc perfusion density**								
Signal strength	8.48 ± 1.24	8.05 ± 1.36	0.213	8.74 ± 1.14	8.28 ± 1.09	0.102	0.423	0.428
Superior	0.43 ± 0.03	0.41 ± 0.03	0.007	0.43 ± 0.03	0.4 ± 0.03	<0.001	0.722	0.303
Inferior	0.43 ± 0.02	0.4 ± 0.04	<0.001	0.44 ± 0.02	0.4 ± 0.03	<0.001	0.073	1.000
Temporal	0.48 ± 0.02	0.46 ± 0.03	<0.001	0.48 ± 0.02	0.46 ± 0.03	0.013	0.320	0.702
Nasal	0.42 ± 0.02	0.42 ± 0.03	0.979	0.44 ± 0.02	0.42 ± 0.02	0.001	0.023	0.469
Outer region mean	0.44 ± 0.02	0.42 ± 0.02	<0.001	0.45 ± 0.02	0.42 ± 0.02	<0.001	0.168	0.616
**Macular ganglion cell-inner plexiform layer thickness (mGCIPL) (μm)**								
Signal strength	8.85 ± 0.99	8.02 ± 1.04	0.001	8.92 ± 0.88	8.31 ± 1.03	0.01	0.768	0.234
Superior	80.81 ± 5.07	70.67 ± 10	<0.001	84.39 ± 5.59	70.78 ± 10.58	<0.001	0.010	0.965
Inferior	75.56 ± 4.69	63.91 ± 8.42	<0.001	81.53 ± 5.32	64.09 ± 10.62	<0.001	<0.001	0.933
Superotemporal	80.15 ± 4.8	70.91 ± 9.07	<0.001	82.26 ± 5.3	67.03 ± 10.61	<0.001	0.104	0.093
Inferotemporal	78.89 ± 4.82	63.47 ± 7.42	<0.001	83.11 ± 5.11	62.59 ± 10.9	<0.001	0.001	0.698
Superonasal	80.96 ± 4.54	73.35 ± 11.74	<0.001	86.24 ± 5.95	76.94 ± 10.64	<0.001	<0.001	0.177
Inferonasal	79.26 ± 5.22	71.02 ± 9.5	<0.001	84.61 ± 5.37	71.66 ± 10.34	<0.001	<0.001	0.784
Average	79 ± 4.79	68.88 ± 6.83	<0.001	83.71 ± 5	68.75 ± 8.92	<0.001	<0.001	0.942
**Peripapillary retinal nerve fiber layer thickness (pRNFL) (μm)**								
Signal strength	7.96 ± 0.94	7.44 ± 1.03	0.003	8.47 ± 0.86	7.75 ± 0.84	0.001	0.027	0.171
Superior	108.33 ±13.29	95.3 ± 19.22	<0.001	127.57 ± 13.81	87.59 ± 17.79	<0.001	<0.001	0.080
Inferior	112 ± 11.24	82.91 ± 14.82	0.001	128.54 ± 14.06	82.41 ± 19.33	<0.001	<0.001	0.899
Temporal	84.74 ± 21.36	69.47 ± 14.42	0.496	72.84 ± 12.8	60.97 ± 14.01	<0.001	0.014	0.013
Nasal	65.52 ± 7.77	67.16 ± 10.83	<0.001	71.26 ± 10.86	64.94 ± 7.44	0.007	0.022	0.321
Average	93.52 ± 5.21	78.74 ± 10.26	0.003	100.53 ± 8.66	73.91 ± 10.14	<0.001	<0.001	0.046

† *P-Value for comparison between control eyes in the highly and the nonhighly myopic groups*.

‡ *P-Value for comparison between glaucomatous eyes in the highly and the nonhighly myopic groups*.

**Table 3:** Area under the receiver operation characteristic curves (AUC) values in highly and non-highly myopic eyes among normal and glaucomatous eyes.

	**Highly myopic**	**Non-highly myopic**	
	**AUC (95%CI)**	**AUC(95%CI)**	***P***
**Optic disc perfusion density**			
Superior	0.725(0.595–0.854)	0.891(0.814–0.967)	0.031
Inferior	0.808(0.704–0.913)	0.938(0.885–0.992)	0.030
Temporal	0.780(0.658–0.903)	0.865(0.775–0.955)	0.275
Nasal	0.646(0.511–0.781)	0.886(0.804–0.968)	0.003
Outer region mean	0.793(0.680–0.906)	0.941(0.890–0.993)	0.019
**Macula perfusion density**			
Outer superior	0.825(0.726–0.925)	0.886(0.802–0.969)	0.360
Outer inferior	0.917(0.841–0.993)	0.929(0.864–0.994)	0.813
Outer temporal	0.766(0.651–0.881)	0.863(0.774–0.952)	0.188
Outer nasal	0.791(0.679–0.903)	0.847(0.753–0.942)	0.447
Inner superior	0.721(0.597–0.845)	0.846(0.749–0.942)	0.119
Inner inferior	0.745(0.625–0.865)	0.850(0.755–0.945)	0.178
Inner temporal	0.734(0.615–0.853)	0.842(0.745–0.940)	0.167
Inner nasal	0.727(0.598–0.856)	0.843(0.747–0.940)	0.157
Inner mean	0.724(0.601–0.846)	0.844(0.747–0.941)	0.131
Outer mean	0.913(0.843–0.983)	0.913(0.849–0.977)	0.997
Full mean	0.845(0.750–0.940)	0.878(0.797–0.959)	0.605
**Peripapillary retinal nerve fiber layer thickness (pRNFL) (μm)**			
Superior	0.767(0.656–0.879)	0.979(0.953–1.000)	<0.001
Inferior	0.946(0.899–0.994)	0.983(0.962–1.000)	0.165
Temporal	0.798(0.687–0.908)	0.908(0.839–0.977)	0.096
Nasal	0.663(0.537–0.789)	0.884(0.807–0.961)	0.003
Average	0.915(0.849–0.980)	0.993(0.982–1.000)	0.021
**Macular ganglion cell-inner plexiform layer thickness (mGCIPL) (μm)**			
Superior	0.856(0.769–0.943)	0.949(0.899–0.999)	0.069
Inferior	0.927(0.861–0.992)	0.952(0.902–1.000)	0.544
Superotemporal	0.868(0.779–0.957)	0.953(0.902–1.000)	0.105
Inferotemporal	0.981(0.951–1.000)	0.959(0.902–1.000)	0.502
Superonasal	0.780(0.674–0.887)	0.883(0.802–0.964)	0.131
Inferonasal	0.826(0.730–0.922)	0.920(0.857–0.984)	0.107
Average	0.921(0.854–0.987)	0.965(0.927–1.000)	0.254

**Table 4:** Comparisons of AUCs of the vessel perfusion density parameters and structural thickness.

	***P*** **–value**	
	**Highly myopic**	**Non-highly myopic**
Optic outer region mean perfusion density vs Average pRNFL thickness	0.068	0.058
Outer inferior macular perfusion density vs Inferotemporal mGCIPL thickness	0.126	0.504
Outer mean macular perfusion density vs Average mGCIPL thickness	0.875	0.168

## Discussion

Retinal vessel density as measured by OCTA was reduced in HM subjects (19, 20). In the meanwhile, pRNFL profile abnormalities also became more prominent with axial elongation in those with high myopia (4, 5, 8). It has been suggested that the peripapillary vessel density changes are useful for diagnosing highly myopic glaucoma (14). In this study, we compared the diagnostic ability of early glaucoma between HM and NHM eyes using OCT and OCTA. We found not only the diagnostic ability of pRNFL thickness, but also the diagnostic ability of peripapillary PD was significantly lower in differentiating highly myopic early glaucoma.

Shin et al. (15) reported that the peripapillary vessel density had a better global and regional correlation with VF mean sensitivity (VFMS) than pRNFL thickness in glaucoma patients with high myopia. They concluded that the peripapillary vessel density may be less affected by myopic changes compared with the pRNFL thickness. Our findings were consistent with their study that we found there was no significant difference in the optic disc perfusion between NHM and HM controls, while the pRNFL thickness in the HM controls was significantly lower than that in the NHM controls. Superficial microvascular network in highly myopic eyes may be stretched rather than lost (19), while glaucomatous vascular damage was found as a dropout of the microvasculature (21). However, Lee et al. (22) later reported that the vessel density was not superior to the pRNFL thickness in the correlation with VFMS when images with segmentation error with improper OCT results were excluded, which implies that as long as a correct and clear OCT image was taken, OCTA did not provide better topographic correlation with VFMS in highly myopic glaucoma patients. Moreover, in Shin's (15) and Lee's (22) study, they included moderate glaucoma (VF defect: 7.5 dB in Shin's study and 9.2 dB in Lee's study), while we included only patients with early glaucoma with VF mean defect <3 MD. To the best of our knowledge, no prior study has compared the diagnostic performance between OCT and OCTA in early glaucoma of highly myopic and non-highly myopic eyes. It is vital that the OCTA diagnostic performance is likely to depend on the severity of glaucoma. Studies have demonstrated that peripapillary vessel density parameters, while comparing with pRNFL thickness, had similar glaucoma diagnostic ability in NHM patients and suggested that the vessel density parameters showed limited clinical value in the early stage (10, 11, 13), which was consistent with our findings in NHM patients. In this study, we further demonstrated that in HM patients, OCTA did not provide better diagnostic ability than conventional structural OCT measurement, whether using the optic nerve head perfusion density or flux index parameter.

The distribution pattern of the pRNFL thickness in HM patients was changed with the superotemporal and inferotemporal RNFL bundles converging temporally. We found the superior, inferior, and nasal quadrants of pRNFL thickness were significantly thinner, while the temporal pRNFL thickness was significantly thicker in HM controls comparing to NHM controls. These findings are in agreement with those from previous studies (4, 8, 23). Akiyasu et al. (8) evaluated patients with similar severity of glaucoma (VF defect: 2.73 dB) as our study population and reported that the abilities of the SD-OCT to detect high myopic early glaucoma were higher if the control group was set with highly myopia comparing to non-highly myopia as the internal normal database in OCT instruments. Their conclusion, along with other studies, suggested OCT instruments should change the internal normal database to increase the glaucoma diagnostic ability in high myopic patients. In this study, we found even the normal controls were set with highly myopia, the glaucoma diagnostic ability was significantly lower in the HM group than in the NHM group whether using average pRNFL or superior thickness. Magnification effect due to longer axial length and difficulty in definition of disc margin due to peripapillary atrophy could not be completely eliminated even using high myopic patients as normal controls.

Ganglion cell complex and macular vessel density measurement showed similar efficiency to detect early glaucoma in NHM patients (12). In our study, the glaucoma diagnostic ability of macular PD and macular GCIPL had no significant difference between HM and NHM eyes. This might be because thinning of the macular inner retinal layer was more supposed to be due to glaucomatous retinal ganglion cell loss, rather than axial length elongation in high myopia (6). Lee et al. (14) had similar findings as ours that the glaucoma diagnostic ability of macular PD and macular GCIPL had no significant difference between HM and NHM patients; however, they further reported that the inferior vessel density ratio had better diagnostic performance comparing to the conventional parameters like inferior RNFL thickness or inferotemporal GCIPL thickness in HM than in NHM patients, which was different from our results. We found inferior macular vessel density and inferotemporal GCIPL thickness had no significant difference in early glaucoma diagnosis whether in HM or NHM patients. There are two points that need to be stressed. First, the highly myopic glaucoma patients in their study were much severer than those in our study in that their VF mean defect was 11.8 MD. Second, we analyzed the macular perfusion density directly provided by the commercial software instead of the vessel density ratio (i.e. outer macular vessel density/inner macular vessel density); hence, our results would be directly applicable to the clinical settings. Third, the sector definitions of radial peripapillary capillaries used in the present study were the ones automatically demarcated by the commercial software of OMAG measurements. However, the ETDRS grid used in Lee's study to define macular and peripapillary sector for perfusion density analysis was primarily developed for the diabetic retinopathy macular evaluation rather than glaucoma evaluation.

Potentially confounding factors (such as IOP, age, and refractive status) may affect vessel density measured by OCTA (9, 24, 25). Higher signal strength was related to higher RNFL thickness (26) and higher macular perfusion density (9) in healthy subjects. Image quality plays an important role in terms of the precision of a measurement. Even within a range of high-quality images, lower signal strength was found to be responsible for the apparent perfusion loss (27). Therefore, we adjusted the signal strength, age, and axial length in the AUC analysis to increase the diagnostic precision in our study.

Several issues need to be considered when interpreting the results of our current study. First, the definition of glaucoma in our study was based on the VF defect, although it did great help in diagnosing early glaucoma with high myopia, our findings might not directly apply to patients with very early preperimetric glaucoma who were not included in our study. Moreover, the results might not be the same in moderate to severe glaucoma. Second, we included only images with signal strength that exceeded the manufacturer's recommended standard. Peripapillary atrophy is common in high myopic eyes, mostly on the temporal side. We excluded peripapillary atrophy in the superior and inferior quadrant extending across from the 3.4-mm-diameter scan circle centered on the optic nerve head by Cirrus HD-OCT in this study. Therefore, the results of this study might only be applicable to eyes with high-quality OCT and OCTA images.

Our study had some limitations. First, because of its retrospective design, we did not investigate the blood pressure of the subjects or their anti-hypertensive medications and we did not measure axial length in advance. Second, the sector definitions of OCT and OCTA measurements used in the present study were the ones automatically demarcated by the software and we did not use other software to redefine the region of interest. It might not be fair to compare it directly since the scan area of vessel density and structural thickness was different; however, our study results provided clinicians the information of those parameters directly provided by commercial software of the Cirrus OCT and OCTA machines which we could easily get in clinical practice.

In conclusion, this study demonstrated that although peripapillary PD and macular PD were significantly reduced in early glaucoma with highly myopia, the diagnostic ability of peripapillary PD in HM patients was significantly lower than that in NHM patients, while macular PD was not. Macular OCTA along with OCT imaging should be included in the imaging algorithm in early glaucoma diagnosis in highly myopic patients.

## Data Availability Statement

The original contributions presented in the study are included in the article/supplementary materials, further inquiries can be directed to the corresponding author.

## Ethics Statement

The studies involving human participants were reviewed and approved by No.21, Sec. 2, Nanya S. Rd. Banciao Dist. Written informed consent for participation was not required for this study in accordance with the national legislation and the institutional requirements.

## Author Contributions

P-YC and J-YW contributed to conceptualization, investigation, writing, reviewing, and editing. J-YW contributed to methodology, validation, and formal analysis. Y-RH contributed to software, validation, and investigation. T-LH contributed to investigation and visualization. P-YC contributed to resources, data curation, writing original draft preparation, visualization, and funding acquisition. J-KW contributed to supervision, investigation, and project administration. All authors have read and agreed to the published version of the manuscript.

## Funding

This project was supported by a grant obtained from the Far Eastern Memorial Hospital (FEMH-2021-C049).

## Conflict of Interest

The authors declare that the research was conducted in the absence of any commercial or financial relationships that could be construed as a potential conflict of interest.

## Publisher's Note

All claims expressed in this article are solely those of the authors and do not necessarily represent those of their affiliated organizations, or those of the publisher, the editors and the reviewers. Any product that may be evaluated in this article, or claim that may be made by its manufacturer, is not guaranteed or endorsed by the publisher.
